# Supplementary material for: A computational approach to quantifying miscounting of radiation-induced double-strand break immunofluorescent foci
Source: Commun Biol. 2022 Jul 14;5:700. doi: 10.1038/s42003-022-03585-5 (PMC9283546; doi:10.1038/s42003-022-03585-5)
Supplement: Supplementary file 2 — Supplemental Material [file 42003_2022_3585_MOESM2_ESM.pdf]

PSF central-axis full width half maximum in μm (X,Y,Z axis)					
	**10x**	**20x**	**40x**	**63x**	**100x**
**Airyscan**	0.72, 0.54, 4.97	0.37, 0.28, 1.7	0.26, 0.18, 0.78	0.25, 0.19, 0.68	0.25, 0.19, 0.61
**gSTED**	–	0.52, 0.51, 1.29*	0.32, 0.5, 0.97	0.2, 0.22, 1.01	0.2, 0.2, 0.84
**Lowlight**	–	0.72, 0.69, 3.68	0.49, 0.43, 1.97	–	0.27, 0.24, 0.74
**MultiPhoton**	1.0, 0.94, 9.9	0.41, 0.43, 2.91**	0.32, 0.34, 0.98	0.23, 0.31, 0.62	–
**Phenix**	–	1.12, 1.14, 3.37	0.56, 0.62, 1.59	0.4, 0.41, 1.42	–
**STED**	–	0.54, 0.45, 9.34	0.26, 0.32, 2.25	0.2, 0.29, 1.32	0.2, 0.2, 0.77

Table S1. The FWHM of the PSF intensity along each central axis. *23x magnification not 20x. **25x magnification not 20x. The STED microscope also has a PSF at 25x which has the following values (0.35,0.38,2.85) for X,Y,Z axis respectively.

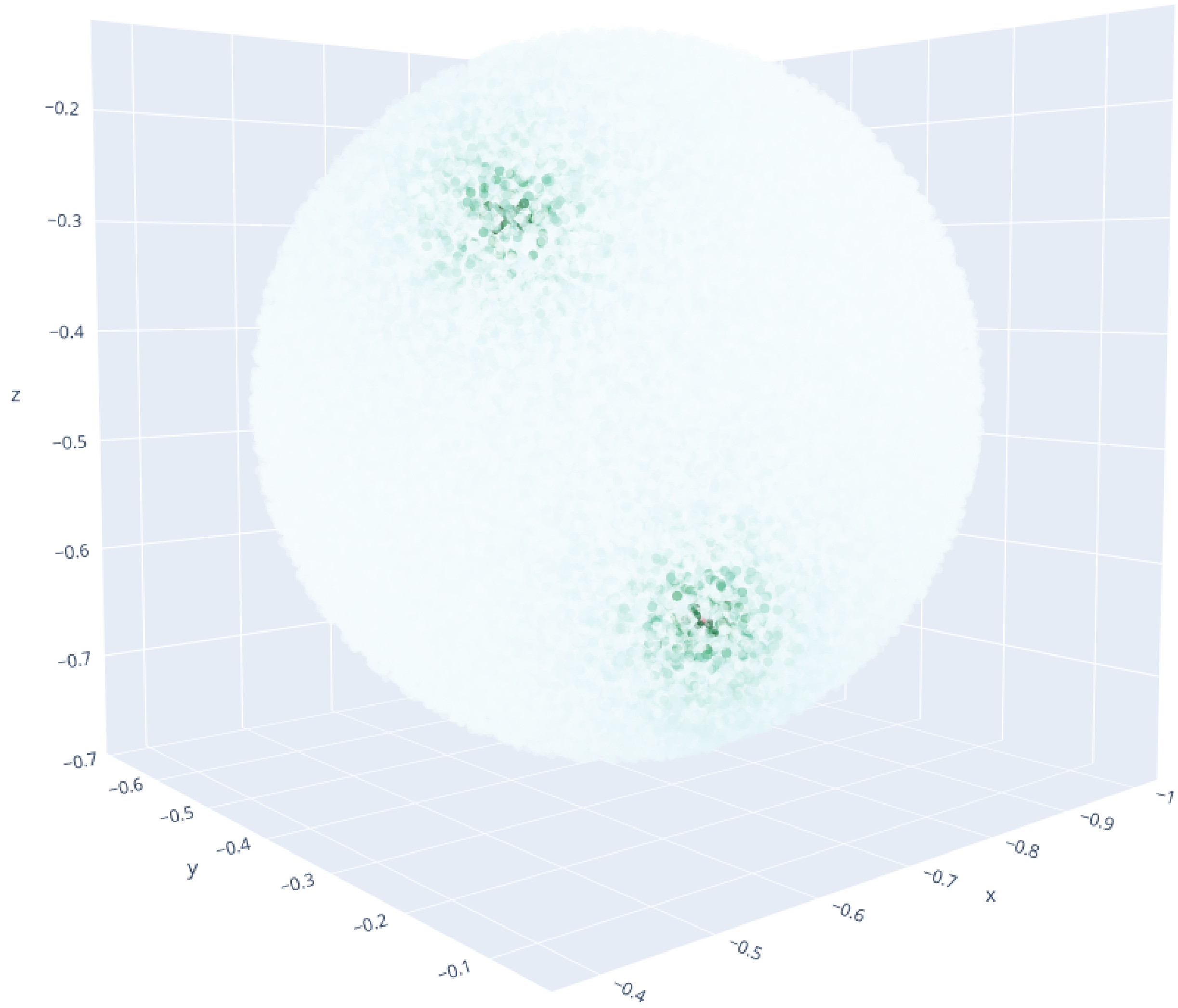

Figure S1. Activation spread of the H2AX histones surrounding two DSBs (red crosses) for a single topological associated domain (TAD) axis units are shown in microns. Each small sphere represents an H2AX histone where no activation is displayed by the sphere being white and activation is displayed as an increasing green colour based on the intensity. Activation values are calculated based on Chip-Seq measurements Arnould et al.,[30] fitted using a Cauchy-Lorentz distribution. The positions of these histones along with the activation value is used by PyFoci in the creation of γ-H2AX marker computational microscope images.

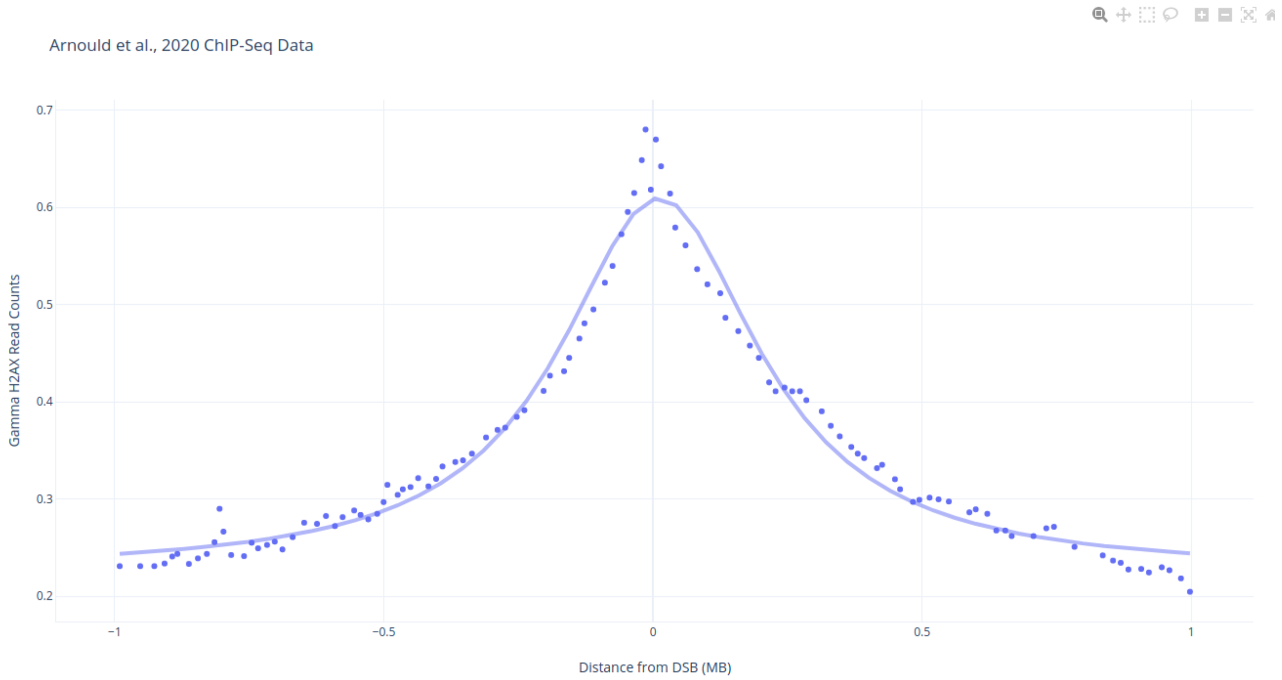

Figure S2. The Cauchy-Lorentz distribution fit (blue-solid line) for γ-H2AX read counts based on the Chip-Seq measurements (blue dots) by Arnould et al.,[30]. This work uses the γ-H2AX read counts as a surrogate for histone activation.

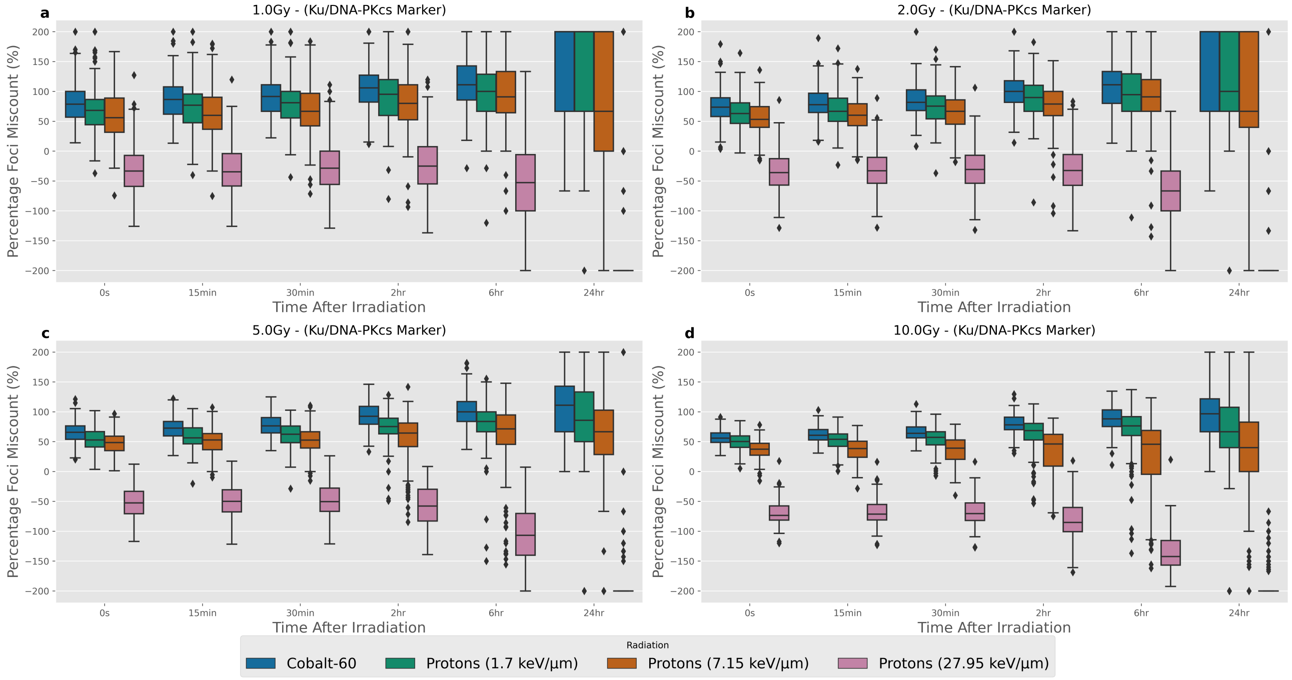

Figure S3. Distributions of the percentage difference between foci detected and the number of DSBs within the central microscope slice of the cell nucleus when using a **Ku/DNA-PKcs marker of fluorescence**. The percentage difference is calculated as the $(counted - simulated/((counted + simulated) * 0.5) * 100$. Values of -200% or +200% were checked and correspond to either foci not being detected due to a low threshold value or at least one break detected when no breaks are present within confocal slice respectively. Error bars correspond to 1.5 times the interquartile range in either direction. Each panel is the same four radiation types and six-time points being compared for different radiation doses, where a, b, c, d correspond to 1.0, 2.0, 5.0, 10.0 Gy respectively. All microscope images have been emulated using the Airyscan 63x point spread function.

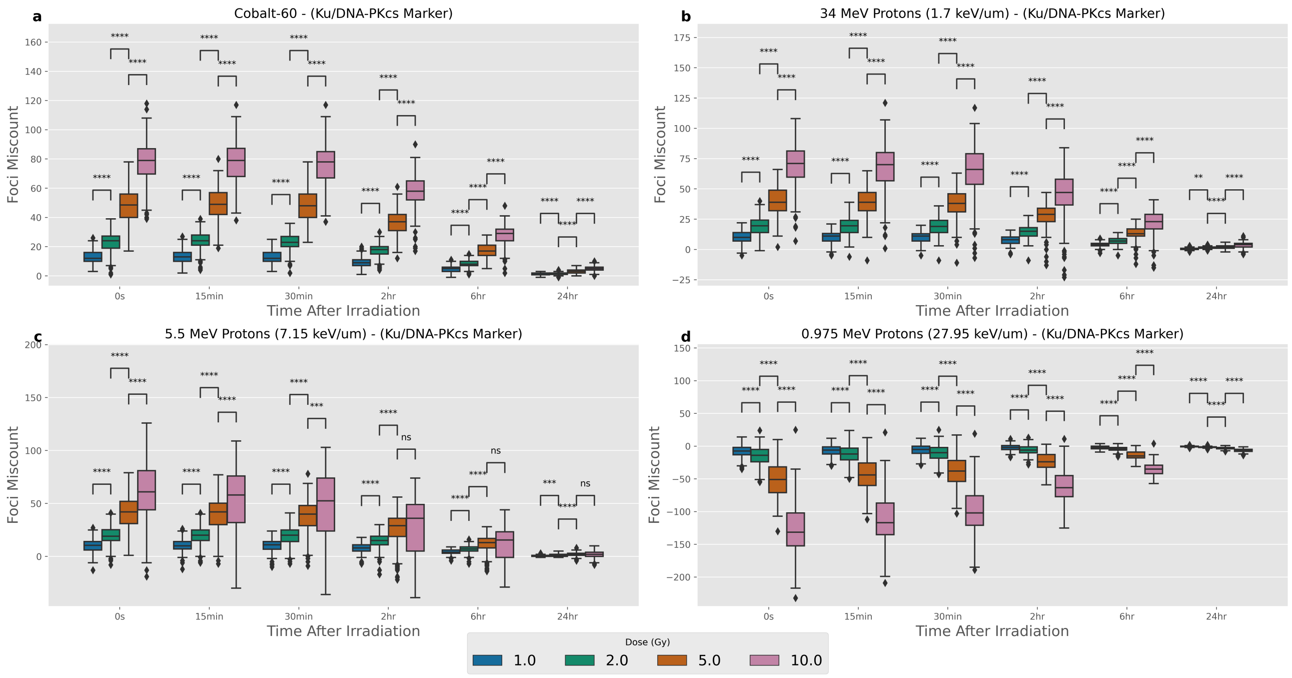

Figure S4. Distributions of miscounting between foci detected and the number of DSBs within the central microscope slice of the cell nucleus when using a **Ku/DNA-PKcs marker of fluorescence**. Each panel is the same four doses and six-time points being compared for different radiation types, where a, b, c, d correspond to Cobalt-60, 34 MeV Protons, 5.5 MeV Protons, 0.975 MeV protons respectively. Error bars correspond to 1.5 times the interquartile range in either direction. Mann-Whitney test between different radiation types at each time point and dose to highlight statistically significant differences. P-values have been adjusted using the Bonferroni correction. Significance notation refers to the following thresholds: ns=P>0.05, *=P>0.01, **=P>1e-3, ***=P>1e-4, ****=P<1e-4. There are 200 samples for each box-plot. All microscope images have been emulated using the Airyscan 63x point spread function.

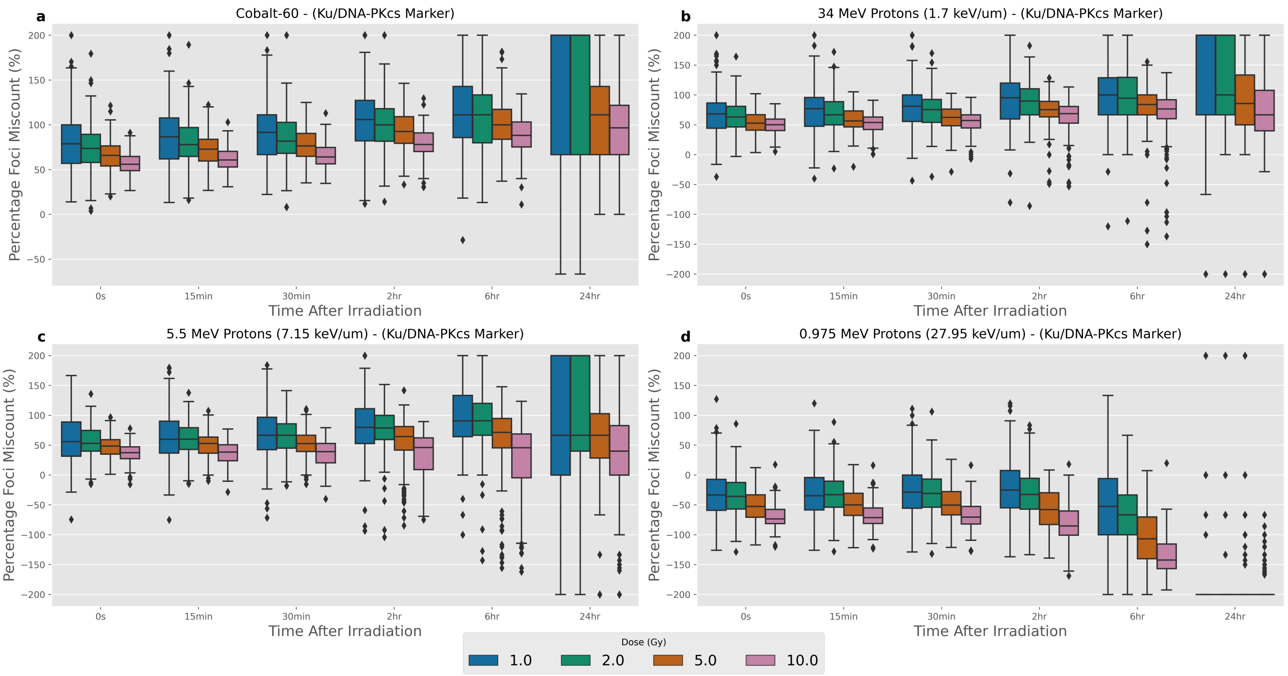

Figure S5. Distributions of the percentage difference between foci detected and the number of DSBs within the central microscope slice of the cell nucleus when using a **Ku/DNA-PKcs marker of fluorescence**. The percentage difference is calculated as the $(counted - simulated/((counted + simulated) * 0.5) * 100$. Values of -200% or +200% were checked and correspond to either foci not being detected due to a low threshold value or at least one break detected when no breaks are present within confocal slice respectively. Error bars correspond to 1.5 times the interquartile range in either direction. Each panel is the same four doses and six-time points being compared for different radiation types, where a, b, c, d correspond to Cobalt-60, 34 MeV Protons, 5.5 MeV Protons, 0.975 MeV protons respectively. All microscope images have been emulated using the Airyscan 63x point spread function.

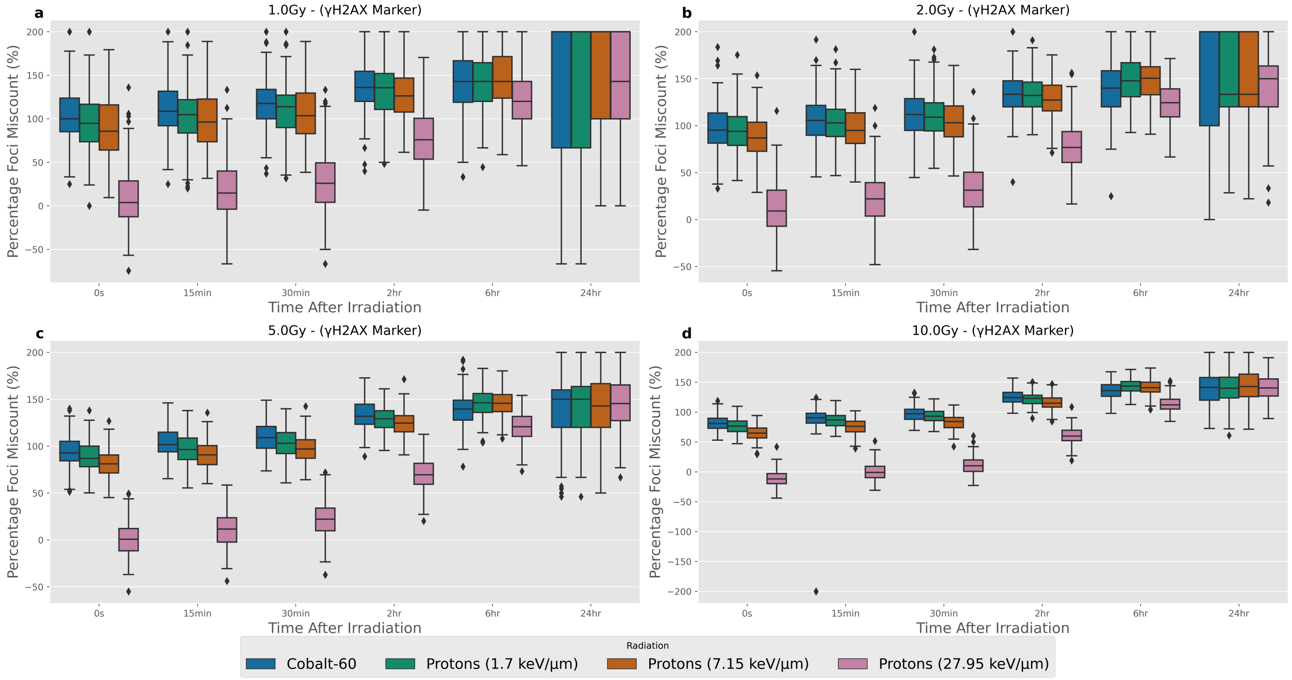

Figure S6. Distributions of the percentage difference between foci detected and the number of DSBs within the central microscope slice of the cell nucleus when using an **γ-H2AX marker of fluorescence**. The percentage difference is calculated as the $(counted - simulated/((counted + simulated) * 0.5) * 100$. Values of -200% or +200% were checked and correspond to either foci not being detected due to a low threshold value or at least one break detected when no breaks are present within confocal slice respectively. Error bars correspond to 1.5 times the interquartile range in either direction. Each panel is the same four radiation types and six-time points being compared for different radiation doses, where a, b, c, d correspond to 1.0, 2.0, 5.0, 10.0 Gy respectively. All microscope images have been emulated using the Airyscan 63x point spread function.

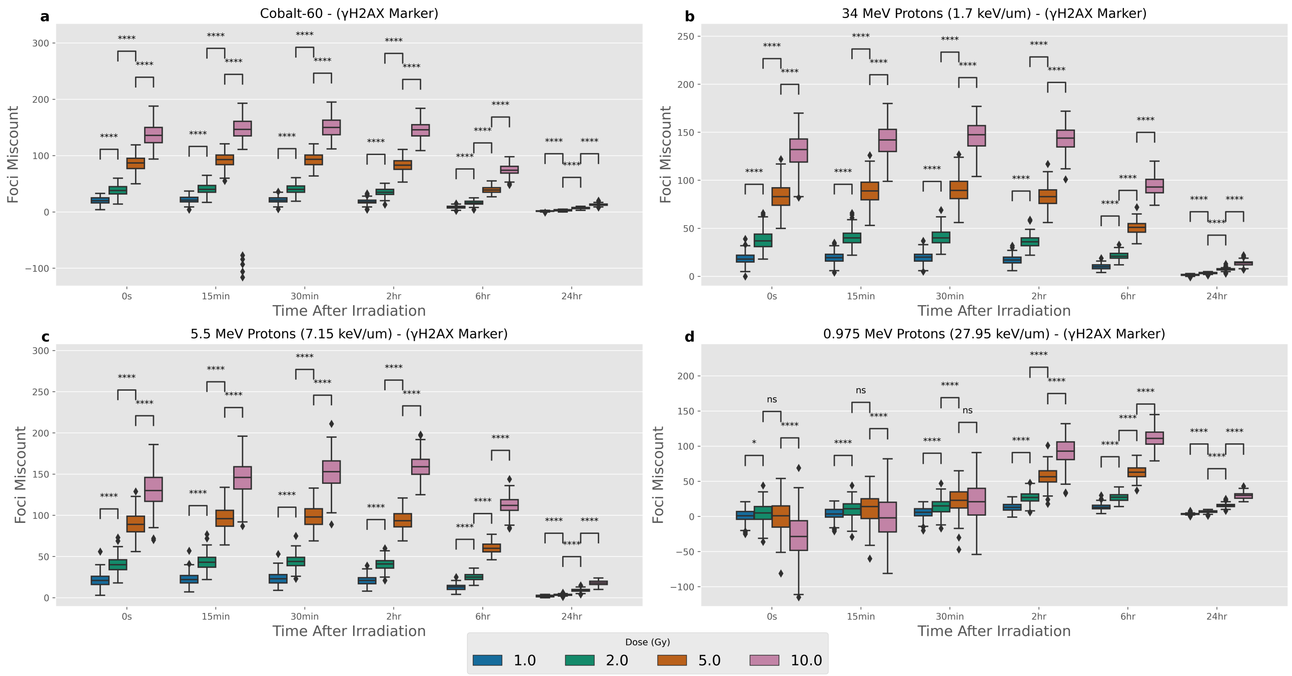

Figure S7. Distributions of miscounting between foci detected and the number of DSBs within the central microscope slice of the cell nucleus when using an **γ-H2AX marker of fluorescence**. Each panel is the same four doses and six-time points being compared for different radiation types, where a, b, c, d correspond to Cobalt-60, 34 MeV Protons, 5.5 MeV Protons, 0.975 MeV protons respectively. Error bars correspond to 1.5 times the interquartile range in either direction. Mann-Whitney test between different radiation types at each time point and dose to highlight statistically significant differences. P-values have been adjusted using the Bonferroni correction. Significance notation refers to the following thresholds: ns=P>0.05, *=P>0.01, **=P>1e-3, ***=P>1e-4, ****=P<1e-4. There are 200 samples for each box-plot. All microscope images have been emulated using the Airyscan 63x point spread function.

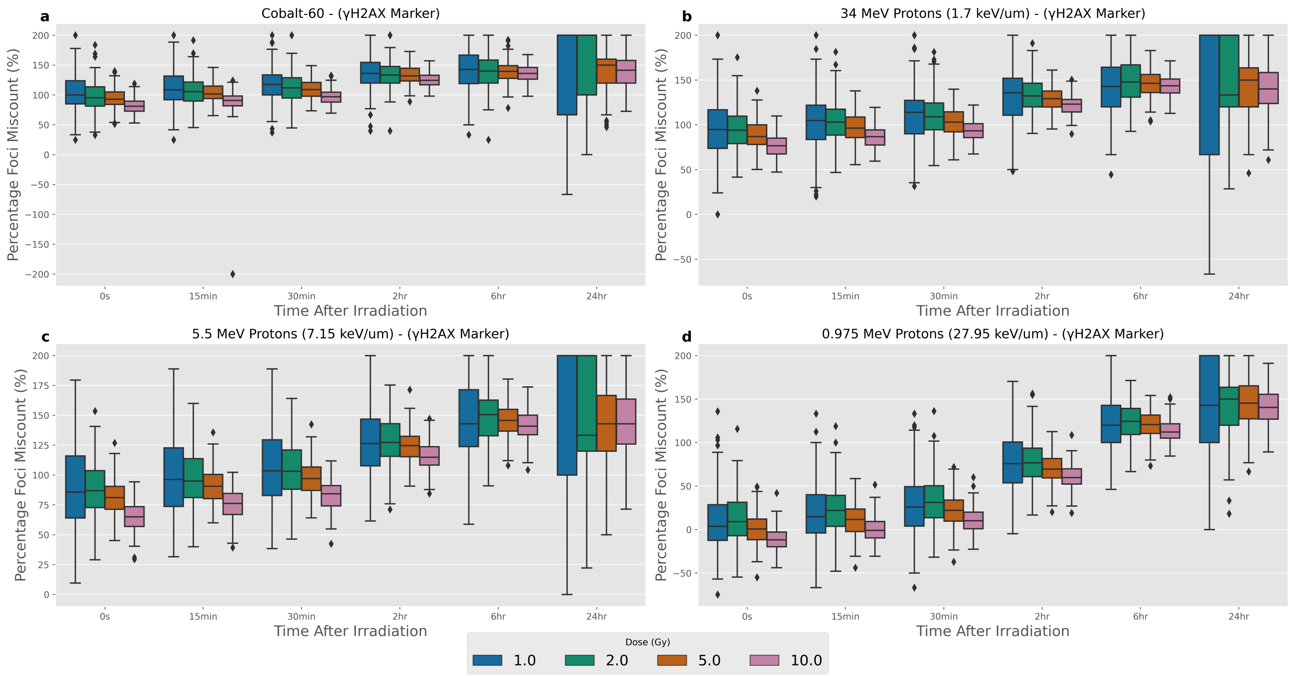

Figure S8. Distributions of the percentage difference between foci detected and the number of DSBs within the central microscope slice of the cell nucleus when using a **γ-H2AX marker of fluorescence**. The percentage difference is calculated as the $(counted - simulated/((counted + simulated) * 0.5) * 100$. Values of -200% or +200% were checked and correspond to either foci not being detected due to a low threshold value or at least one break detected when no breaks are present within confocal slice respectively. Error bars correspond to 1.5 times the interquartile range in either direction. Each panel is the same four doses and six-time points being compared for different radiation types, where a, b, c, d correspond to Cobalt-60, 34 MeV Protons, 5.5 MeV Protons, 0.975 MeV protons respectively. All microscope images have been emulated using the Airyscan 63x point spread function.

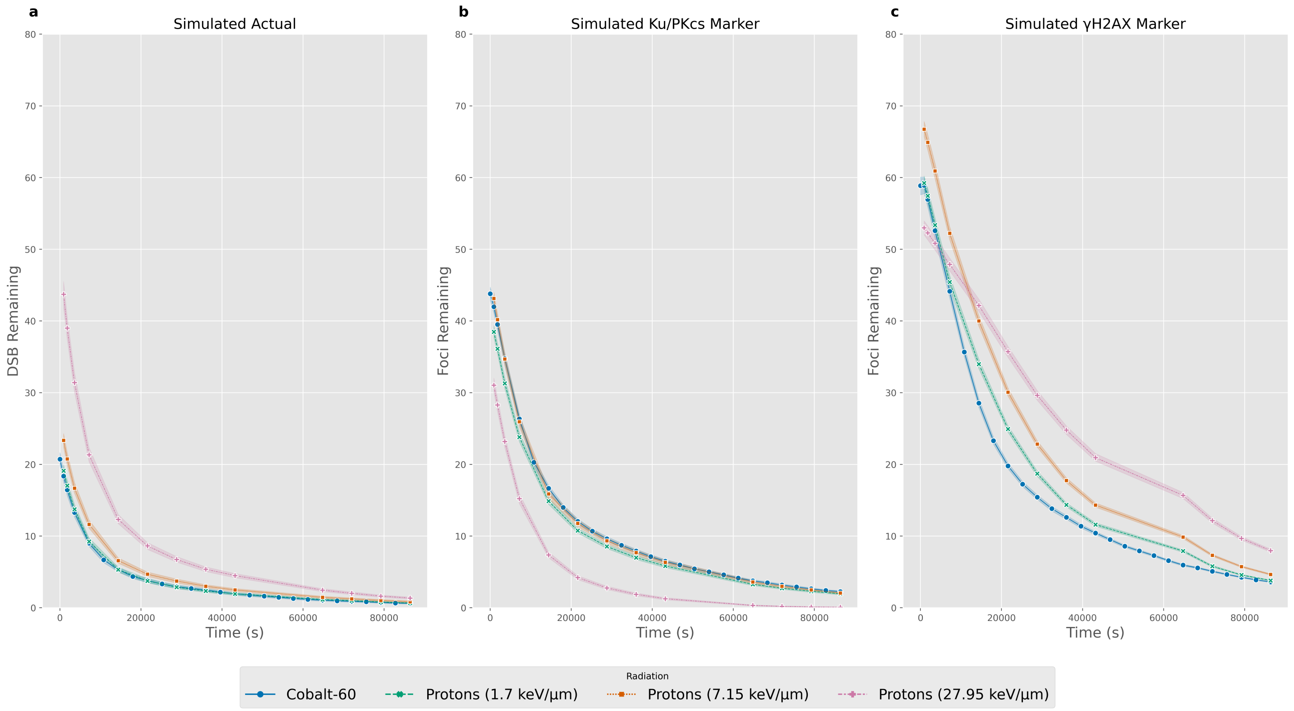

Figure S9. Repair kinetics comparison between cells irradiated with four different incident radiation conditions. a) shows the repair kinetics of the actual number of DSBs within the evaluated microscope slice. The repair kinetics applied is kept constant across all radiation types to separate the impact of miscounting alone. b) the repair kinetics when calculated from the foci detected in the Ku/DNA-PKcs marker microscope images. c) the repair kinetics when calculated from the foci detected in the γ-H2AX marker microscope images. All microscope images have been emulated using the Airyscan 63x and the results shown are for 2Gy of radiation. Error bars are shown as banding and corresponds to the 95% confidence interval.

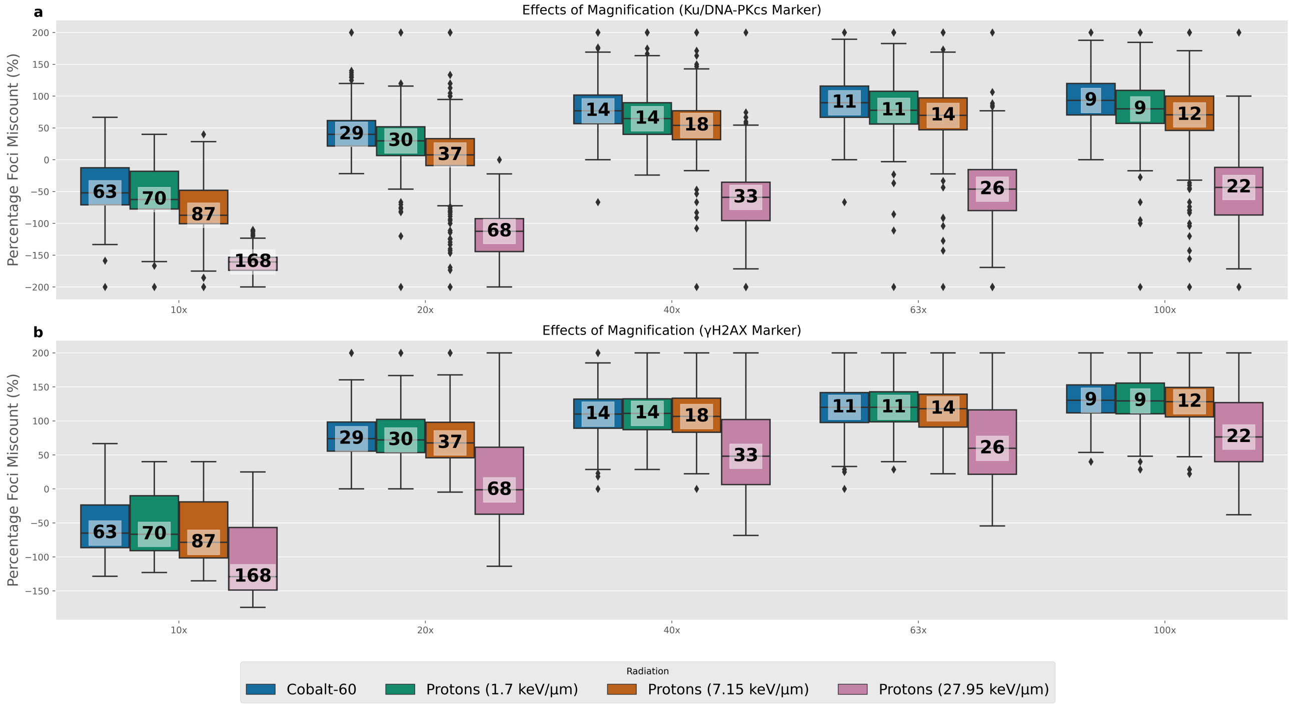

Figure S10. Comparison of percentage miscounting at different Airyscan magnifications. The value shown in white within each box-plot is the actual number of DSBs within the evaluated microscope slice to give perspective on the amount of miscounting each percentage corresponds to. a) are the results when using a Ku/DNA-PKcs marker. b) are the results when using an γ-H2AX marker. All microscope images are at 2 Gy dose and for all the 6 time points (0s, 15mins, 30 mins, 2hrs, 6hrs and 24hrs). Error bars correspond to 1.5 times the interquartile range in either direction. There are 1200 samples for each box-plot.

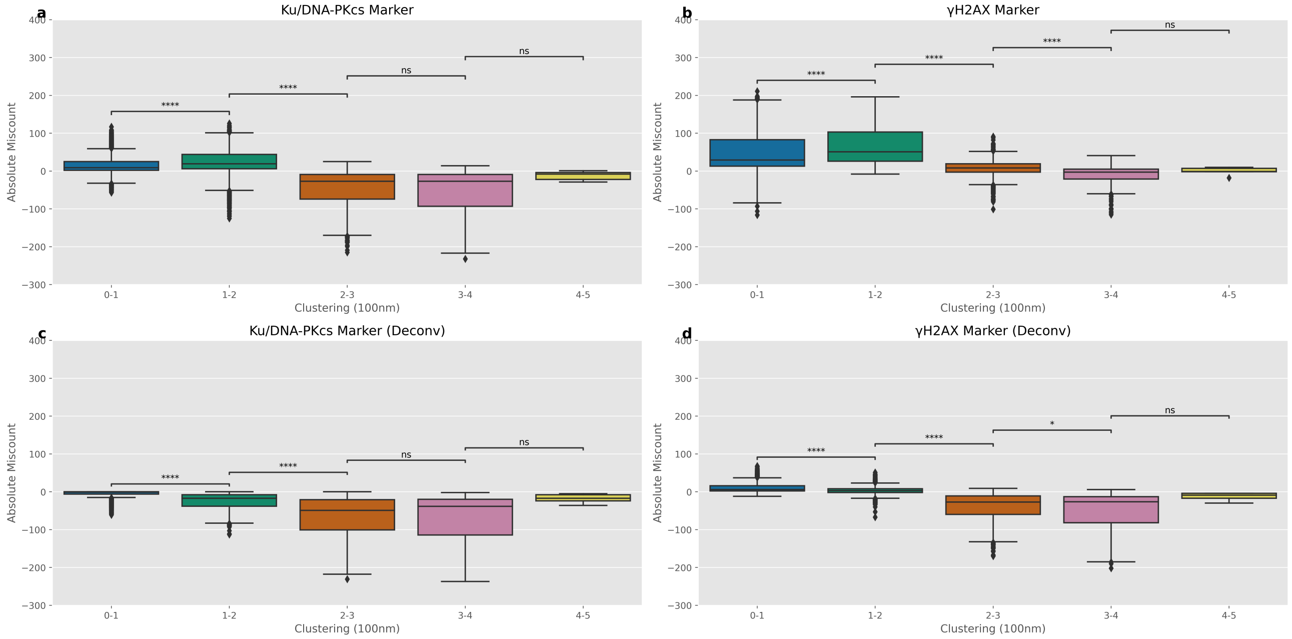

Figure S11. Categorising the magnitude of miscounting as a function of DSB clustering. The values of clustering are calculated based on the known amount of DSBs within the cell nucleus at a given time point. Clustering equates to the average number of DSBs within proximity to each DSB within the simulated cell nucleus. Where proximity for this study is characterised as 100 nm. Clustering is increased for early time points, higher dose, higher LET and is decreased for the opposite. Therefore, the clustering combines time, dose and radiation type parameters into a single metric. Panel a and b are for the DSB and γ-H2AX markers respectively. Panel c and d are for the DSB and γ-H2AX markers with perfect deconvolution respectively. Error bars correspond to 1.5 times the interquartile range in either direction. Mann-Whitney test between categorised clustering values to highlight statistically significant differences. P-values have been adjusted using the Bonferroni correction. Significance notation refers to the following thresholds: ns=P>0.05, *=P>0.01, **=P>1e-3, ***=P>1e-4, ****=P<1e-4. All microscope images have been emulated using the Airyscan 63x point spread function.

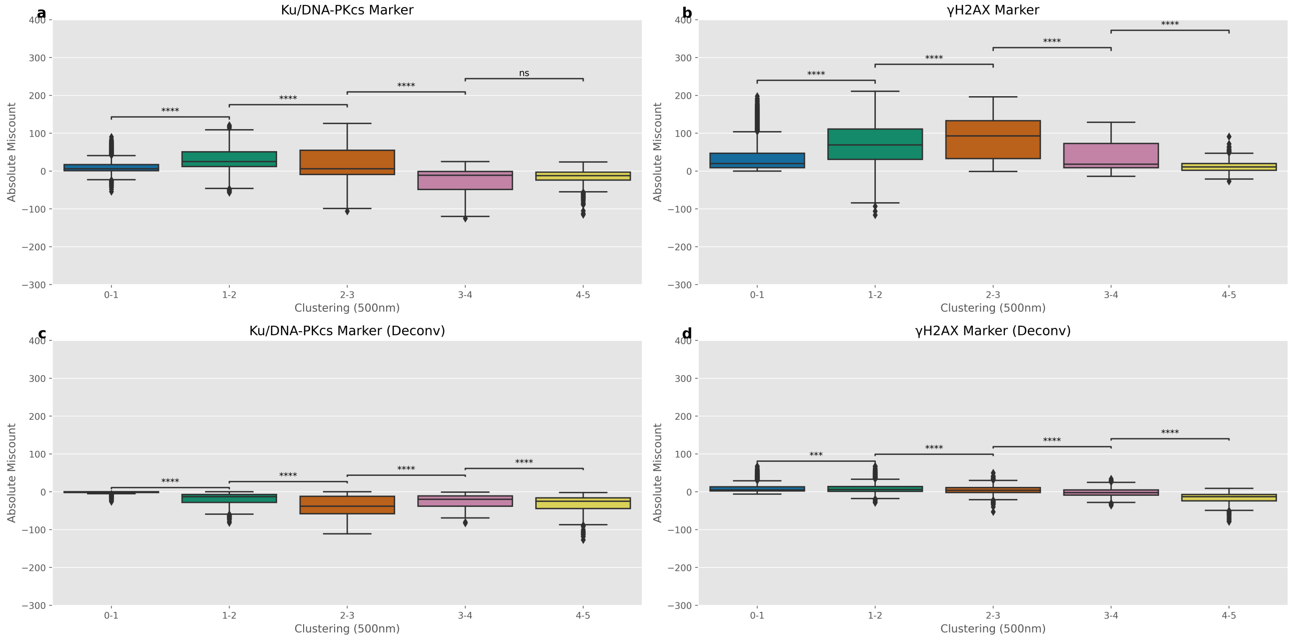

Figure S12. Categorising the magnitude of miscounting as a function of DSB clustering. The values of clustering are calculated based on the known amount of DSBs within the cell nucleus at a given time point. Clustering equates to the average number of DSBs within proximity to each DSB within the simulated cell nucleus. Where proximity for this study is characterised as 500 nm. Clustering is increased for early time points, higher dose, higher LET and is decreased for the opposite. Therefore, the clustering combines time, dose and radiation type parameters into a single metric. Panel a and b are for the DSB and γ-H2AX markers respectively. Panel c and d are for the DSB and γ-H2AX markers with perfect deconvolution respectively. Error bars correspond to 1.5 times the interquartile range in either direction. Mann-Whitney test between categorised clustering values to highlight statistically significant differences. P-values have been adjusted using the Bonferroni correction. Significance notation refers to the following thresholds: ns=P>0.05, *=P>0.01, **=P>1e-3, ***=P>1e-4, ****=P<1e-4. All microscope images have been emulated using the Airyscan 63x point spread function.
